# Research Progress in the Interconversion, Turnover and Degradation of Chlorophyll

**DOI:** 10.3390/cells10113134

**Published:** 2021-11-12

**Authors:** Xueyun Hu, Tongyu Gu, Imran Khan, Ahmad Zada, Ting Jia

**Affiliations:** 1 Joint International Research Laboratory of Agriculture and Agri-Product Safety of the Ministry of Education of China, Yangzhou University, Yangzhou 225009, China; xyhulab@yzu.edu.cn; 2 Key Laboratory of Plant Functional Genomics of the Ministry of Education, Yangzhou University, Yangzhou 225009, China; 3 College of Bioscience and Biotechnology, Yangzhou University, Yangzhou 225009, China; mz120191171@yzu.edu.cn (T.G.); dh18006@yzu.edu.cn (I.K.); dh19025@yzu.edu.cn (A.Z.)

**Keywords:** chlorophyll cycle, chlorophyll turnover, chlorophyll degradation, pathway, enzymes

## Abstract

Chlorophylls (Chls, Chl *a* and Chl *b*) are tetrapyrrole molecules essential for photosynthetic light harvesting and energy transduction in plants. Once formed, Chls are noncovalently bound to photosynthetic proteins on the thylakoid membrane. In contrast, they are dismantled from photosystems in response to environmental changes or developmental processes; thus, they undergo interconversion, turnover, and degradation. In the last twenty years, fruitful research progress has been achieved on these Chl metabolic processes. The discovery of new metabolic pathways has been accompanied by the identification of enzymes associated with biochemical steps. This article reviews recent progress in the analysis of the Chl cycle, turnover and degradation pathways and the involved enzymes. In addition, open questions regarding these pathways that require further investigation are also suggested.

## 1. Introduction

Chlorophyll (Chl), the most abundant pigment existing in the photosynthetic system of land plants and algae, is an indispensable pigment for absorbing light energy and transferring electrons during photosynthesis. It is estimated that approximately 1.2 billion tonnes of Chl is seasonally synthesised and degraded on earth every year [1]. The colour change in leaves and fruits from green to yellow or red in autumn is the most conspicuous impression of losing Chl. Nevertheless, Chl is a potential cellular phytotoxin, and excess Chl and its derivatives must be rapidly degraded. Otherwise, cell death will occur via reactive oxygen species (ROS) generated by excess Chl when Chl degradation is inhibited [2,3,4]; thus, Chl metabolism is strictly regulated during different phases of plant development. At the same time, total Chl per leaf area is one of the factors that showed most responsiveness to nutrient availability and different environmental stresses, highlighting the paramount role of antenna size regulation in plant acclimation [5]. For example, Chl content increases under salinity and seasonality, whereas it decreases under chilling, ozone and drought [6]. Plants contain Chl *a* and *b*, and the ratio of Chl *a* to *b* in the leaves is changeable to adapt to variable light intensities to a certain degree. Chl *a*/*b* ratio could be an indicator of the intensity of irradiation acclimation and the structure of the photosynthetic apparatus [6]. The interconversion system between Chl *a* and *b* refers to the Chl cycle. When photosystem (PS) damage occurs during stress conditions, PSII repair and Chl turnover subsequently occurs. A Chl turnover pathway is required for PSII repair and protection of young leaves from photodamage [7]. During leaf senescence or fruit maturation, Chl degradation is the major Chl metabolism direction. Chl and proteins are degraded and replaced, and movable nutrients are released and rapidly transferred to developing organs to facilitate plant growth and development. Moreover, Chl degradation is an important survival strategy for removing the phytotoxin pigments from chloroplasts and resistant to biotic and abiotic stresses [8].

In recent decades, Chl metabolic pathways and the key enzymes in Chl metabolic flow have been well established and identified. Chl biosynthesis is essential for photosynthesis, and it has been well studied and reviewed by Qiu et al. [9]. However, uncovering the Chl cycle, turnover and degradation pathway is a more recent research topic, and questions remain. It is widely accepted that the identification of the structure of nonfluorescent Chl catabolites (NCCs) is a hallmark study to break through the “biological enigma” existing in the Chl degradation pathway [10]. Recent studies have shown that dioxobilin-type nonfluorescent Chl catabolites (DNCCs), rather than NCCs, are the major degradation products of Chl in some senescent leaves [11]. The cytochrome P450 monooxygenase CYP89A9, which is localised outside chloroplasts, is responsible for the accumulation of DNCCs in senescent *Arabidopsis* leaves [12]. More recently, three revolutionary discoveries made great contributions to uncovering Chl turnover and degradation pathways. One is that Mendel’s green cotyledon gene (*SGR*) encodes magnesium dechelatase, which is responsible for extracting magnesium (Mg^2+^) from Chl *a* [13]. The second is a novel Chl dephytylase gene called CLD1, which works to cleave the phytol chain from Chl *a* [14]. The third is TRANSLOCON AT THE INNER CHLOROPLAST ENVELOPE55 (TIC55), which is localised in the envelope and plays a role in phyllobilin hydroxylation [15]. Elucidation of SGR and TIC55 completely integrates the missing linkage in the Chl breakdown pathways, and the function of CLD1 provides neoteric insight into enzymatic activity during Chl turnover [14]. Here, we review the new evolution of the Chl cycle, turnover and degradation pathways, including the biochemical steps, enzymes involved, regulatory mechanisms and remaining questions.

## 2. Chl Cycle

In higher plants, Chl *a* exists in both core complexes and light harvesting complexes (LHC), while Chl *b* exclusively exists in LHC [16]. Chl *a* is essential for the photochemistry, while Chl *b* provides plants an advantage in harvesting a wider range of light because Chl *b* has a strong absorption around 450 nm, which is a region of light that Chl *a* does not efficiently absorb. Therefore, Chl *b* is highly significant in increasing harvesting of light [17]. Moreover, the biosynthesis and breakdown of Chl *b* is tightly linked with the construction and destruction of LHC, which can be adjusted under various light conditions [18]. For example, when high-light-grown plants are transferred to low-light conditions, the amount of LHCII/core complexes of the PSII ratio increases and more Chl *b* accumulates, indicating an increment in the antenna size of PSII for harvesting more light for photosynthesis. On the contrary, when low-light-grown plants are transferred to high-light conditions, the amount of LHCII/core complexes of the PSII ratio decreases, and less Chl *b* accumulates. Thus, it can reduce the photoinhibition of photosynthesis by excessive light. In addition, PSI/PSII ratio is also regulated by Chl *a* and Chl *b* interconversion, revealed by the analysis of Chl *b* deficient mutants [19]. Chl *b*-deficient wheat mutant lines had a lower content of photo-oxidizable PSI and lower PSI/PSII ratio, which limits the photoprotection of PSI and PSII in early growth stages [19,20]. Therefore, Chl *b* metabolism forms an essential part of light acclimation mechanisms in plants.

In the Chl biosynthesis pathway, chlorophyllide (Chlide) *a* is the immediate precursor of Chl *a* [21]. It has been proposed that Chlide *a* is also the immediate precursor of Chlide *b* because Chlide *a* oxygenase (CAO) can catalyse Chlide *a* to Chlide *b in vitro* [22]. If this pathway is true for Chl *b* biosynthesis, this suggests that Chlide *b* is subsequently ligated with phytol side chains to form Chl *b* by the catalysis of Chl synthase (CHLG) [23]. Although CAO cannot react with Chl *a in vitro*, it has been proposed that CAO reacts with Chl *a in vivo*. This is because Chl *a* did not react with CAO in the *in vitro* experiments mentioned above due to its hydrophobic nature. Chl *a* binds to proteins *in vivo* to which CAO is accessible. Thus, it is possible that CAO may be able to react with Chl *a* [9]. This hypothesis is supported by the discovery of the conversion of Chl *a* to Chl *b* when the *de novo* synthesis of Chl was stopped under darkness [24,25]. Further studies are necessary to clarify the pathway for Chl *b* biosynthesis from Chl *a* or Chlide *a*.

CAO is the sole discovered enzyme that catalyses the conversion of Chl(ide) *a* to Chl(ide) *b* [26]. CAO is a member of the Rieske nonheme iron oxygenase family and can carry out two successive oxygenation reactions [22]. The regulation role of CAO has been extensively studied. The amino acid sequence of CAO in higher plants consists of A-, B- and C-domains, while only the conserved C-domain was found in the CAO of *Prochlorothrix hollandica*, a cyanobacterium. The C-domain catalyses Chl *b* synthesis [27], the A-domain is responsible for degrading excess CAO by combining with Clp proteases (ubiquitous AAA+ family of ATPase caseinolytic proteases), and the B-domain is a linker between the A- and C-domains [28]. When full-length CAO, including the A-, B- and C-domains, was expressed, transgenic plants did not accumulate CAO protein as well as wild-type plants, and the Chl *b* content was also similar to that in wild-type plants. Transgenic plants expressing truncated CAO without the A-domain accumulated high levels of protein and exhibited significantly high amounts of Chl *b*. This means that the A-domain is essential to maintain a low level of CAO to avoid excess Chl *b* synthesis. The abundance of CAO is regulated by negative feedback from Chl *b* [29]. The CAO mRNA level also correlates with the Chl *a*/*b* ratio in wild-type *Arabidopsis thaliana* [30]. Illumination intensity affects *CAO* mRNA abundance and the Chl *a*/*b* ratio [31]. When *Arabidopsis* was transferred from low-light to high-light conditions, the CAO mRNA level decreased, which was associated with an increase in the Chl *a*/*b* ratio. In contrast, the Chl *a*/*b* ratio decreased. These results indicate that the transcriptional level of CAO partially controls the synthesis of Chl *b* in light acclimation. A recent study showed that the expression of CAO can not only enlarge the antenna size of the PS but also balance the energy transfer between PSI and PSII by catalysing Chl *b* synthesis [32]. Defects in the *PGL* (*pale green leaf*) gene, which encodes CAO in rice, exhibit premature senescence of leaves under both natural and dark-induced conditions, enhance the accumulation of ROS and indirectly affect the rice grain yield [33].

Chl *b* to Chl *a* conversion also occurs in plants, especially during Chl degradation. *Arabidopsis* has two isozymes of Chl *b* reductase (CBR), NON-YELLOW COLORING 1 (NYC1) and NYC1-like (NOL) [34,35]. Both of these enzymes are responsible for degrading Chl *b* to seven-hydroxymethyl-Chl *a* (HMChl *a*) in the Chl cycle. The activities of NYC1 and NOL are different in rice and *Arabidopsis*. Mutants of *nyc1* or *nol* in rice showed a stay-green phenotype and high levels of retained Chl *b* and LHCII [34,36]. NYC1 and NOL may form a complex that acts as a CBR, as suggested by the physical interaction of NYC1 and NOL *in vitro*. However, in *Arabidopsis*, degradation of Chl *b* is not significantly affected by loss of NOL, implying that NYC1 is the main enzyme that regulates Chl *b* levels [35]. However, the peripheral antenna size of PS II in NOL-overexpressing plants was also observed to be smaller than that in wild-type plants [37]. In addition to the effect on leaves, CBR-lacking *Arabidopsis* mutants exhibit reduced seed longevity and germination because a large amount of Chl is retained [38].

Seven-hydroxymethyl chlorophyll *a* reductase (HCAR) is demonstrated to be the enzyme that catalyses the second step of Chl *b* to Chl *a* reduction: the conversion from HMChl *a* to Chl *a* [39]. HCAR consists of a flavin adenine dinucleotide and an [4Fe-4S] iron-sulfur centre as cofactors, and phylogenetic analysis revealed that the evolution of HCAR was from divinyl Chlide vinyl reductase, an enzyme involved in Chl biosynthesis [39]. A mutant lacking HCAR was found to accumulate HMChl *a*, and a surprising amount of pheophorbide *a* (Pheide *a*, a Mg^2+^- and phytol-free intermediate of Chl *a* breakdown) was found in dark-induced senescent intact plants. Neither HMChl *a* nor Pheide *a* was detected in *hcar-1*/*nyc1*/*nol* triple mutant plants after dark-induced senescence [39]. These results suggest that the accumulation of Pheide *a* in the dark-induced senescent leaves of *hcar* mutants requires the accumulation of HMChl *a*. Recently, dark-induced detached leaves of *HCAR* knockout mutants were found to turn yellow as well [40]. In addition, the natural senescence of these mutant plants showed no distinguishing senescent phenotype from that of wild-type plants. The results suggested that HCAR is not essential for leaf senescence [40]. Another pathway may exist for Chl *b* degradation to bypass HCAR. For example, HMChl *a* is directly degraded without being converted to Chl *a*.

**Figure 1:**
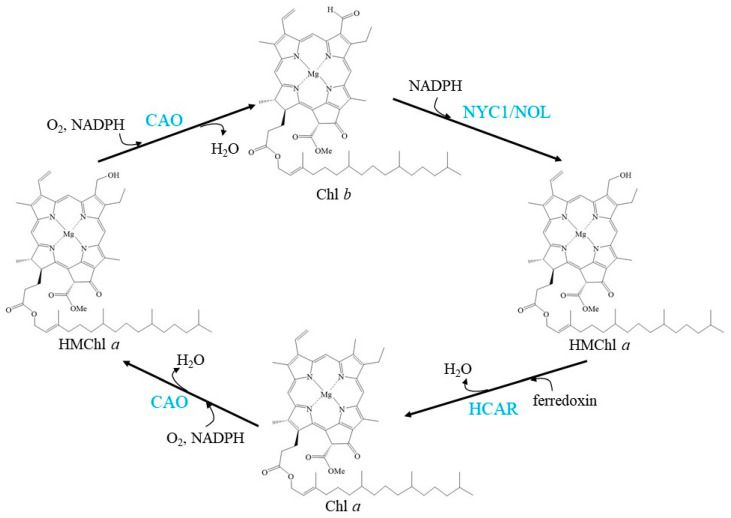
Schemes of the enzymatic reactions in Chl cycle. Blue characters indicate the enzymes.

CAO, NYC1/NOL, and HCAR regulate the balance of the Chl cycle by interconversion between Chl *a* and Chl *b*, exhibiting a fluctuating Chl *a*/*b* ratio (Figure 1).

The Chl *a*/*b* ratio must be finely regulated to adapt to light conditions. Overexpressing A-domain-deleted CAO causes Chl *b* overaccumulation and a much lower Chl *a*/*b* ratio, which subsequently leads to the accumulation of NYC1 [41]. Chl *b* feedback to CAO and feedback to NYC1 in the Chl cycle has been suggested to be an indispensable mechanism for the monitoring and control of the light-harvesting apparatus.

## 3. Chl Turnover (Salvage Cycle)

**Figure 2:**
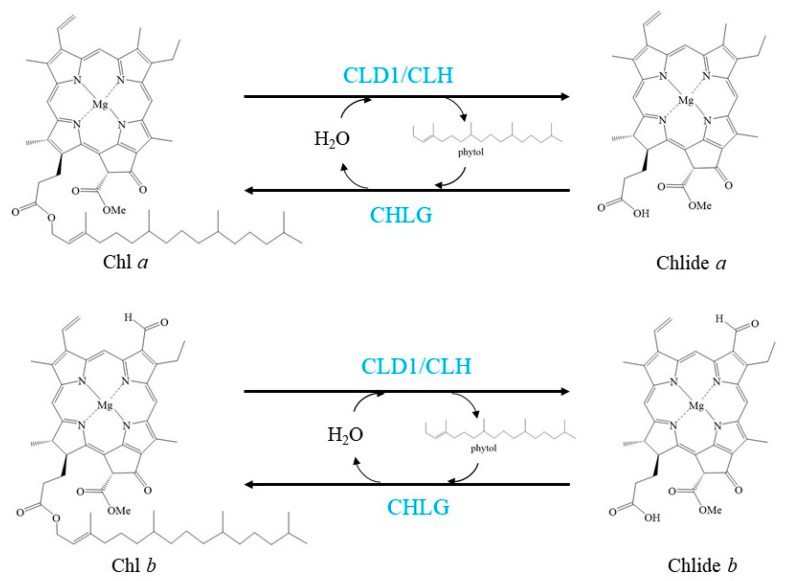
Schemes of the enzymatic reactions in Chl turnover. Blue characters indicate the enzymes.

Chl has also been considered to be turned over and transformed in green leaves, as demonstrated by the pulse-chase isotope labelling of Chl in green leaves of several plant species [14,42,43,44]. These labelling experiments also uncovered that Chl turnover mainly affects Chl *a*. Chl turns over via de- and rephytylation in green plant organs is coupled to the repair of damaged PSII [14] (Figure 2).

D1 protein is the PSII reaction center; however, it is unstable and is subject to damage by irradiation and heat stress [45,46]. The damaged D1 should be degraded by proteases, and a new D1 protein must be subsequently inserted into the partially disassembled PSII, which is known as PSII repair [47,48]. There are three Chl *a*, and one pheophytin *a* (Phetin *a*) binds to each D1 protein; therefore, these pigments need be released from damaged D1 proteins before these D1 proteins are degraded, and they need bind to new D1 proteins before these D1 proteins are inserted to PSII during PSII repair. In this process, Chl *a* and Phetin *a* undergo turnover other than de novo biosynthesis, which is important for repairing PSII immediately to respond to the rapid change of environment, such as light and temperature.

Lin et al. reported a novel enzyme that can dephytylate Chl to produce Chlide, and this enzyme was named Chl dephytylase1 (CLD1) [14]. CLD1 is conserved in oxygenic photosynthetic organisms. It belongs to the α/β-hydrolase superfamily and shares structural similarity with pheophytinase (PPH), another enzyme involved in Chl degradation during leaf senescence [49]. CLD1 is predominantly expressed in green organs but not in the senescent phase. A new version of CLD1, *cld1-1*-encoded enzyme, possesses significantly higher activity than the wild-type enzyme [14]. The *cld1-1* mutant and CLD1-overexpressing seedlings proportionally accumulated Chlide derived from Chl dephytylation after heat shock, which resulted in light-dependent cotyledon bleaching. Reducing CLD1 expression diminished thermotolerance and the photochemical efficiency of PSII under prolonged moderate heat stress [14]. Taken together with the observation that the increased or decreased activity of CLD1 never affects Chl breakdown in dark-induced senescent leaves, CLD1 has been suggested to be the long-sought Chl dephytylation enzyme active during Chl turnover at steady state [14]. Further study is required to demonstrate the mechanism of CLD1 involved in dephytylation of Chl from D1 in the PSII repair cycle.

CHLG is an enzyme that rephytylates Chlide *a* to form Chl *a* under heat shock [23]. In this step, the substrate Chlide *a* is from de-esterified Chl *a* [14,23]. Under nonstress conditions, *chlg-1* mutant seedlings accumulated higher levels of Chlide *a*, suggesting that CHLG is also involved in Chl turnover under nonstress conditions. Interestingly, *chlg-1* mutant seedlings accumulated Chlide *a* but did not accumulate Chlide *b* after heat stress. The results support previous findings that Chl turnover mainly affects Chl *a* [42,43]. Thus, CLD1 and CHLG form a Chl salvage cycle for Chl turnover, and CLD1/CHLG in this cycle should maintain dynamic equilibrium to avoid overaccumulation of Chlide *a*, which is a phototoxic intermediate [14].

Recently, chlorophyllase 1 (CLH1), one isoform of the first identified enzyme that can catalyse the dephytylation of Chl, was discovered to protect young leaves from long-term photodamage by facilitating filamentous temperature-sensitive H protease (FtsH)-mediated D1 degradation in PSII repair [7]. It is interesting that CLH1 is localised at the thylakoid membrane of developing chloroplasts in young leaves, while it is localised outside the developed chloroplasts in mature leaves [7,50,51]. Although both CLHs and CLD1 are able to cleave the ester bond of Chls, the catalytic efficiency of CLHs is much higher than that of CLD1 [7,52]. In addition, CLH is the only enzyme shown to dephytylate Phetins *in vitro* [53]. Study on Phetins turnover is lacking. It is interesting to demonstrate that whether Phetins undergo de- and rephytylation is coupled to the PSII repair cycle and which enzymes catalyse these two reactions. It is uncertain why there are two enzymes, CLHs and CLD1, function in the dephytylation of Chl during Chl turnover. One possibility is that CLHs are required for Chl turnover only in developing chloroplasts, while CLD1 functions in all developmental stages of chloroplasts. This is because CLHs are not located in mature chloroplasts of *Arabidopsis* leaves [7,50,51]. Another possibility is that these two enzymes play roles in different conditions. For example, CLHs work under high light, while CLD1 works under heat shock conditions. Further investigation is also needed to determine whether enzyme(s) other than CHLG is(are) involved in Chl rephytylation during Chl turnover in developing chloroplasts. Although Chl turnover is coupled to the repair cycle of PSII, plants lacking CLD1 or CLHs did not show obvious phenotype under normal growth conditions [23,50]. It is interesting to investigate the effect of lacking both CLD1 and CLHs to the PS of plants.

## 4. Chl Degradation

Chl degradation is an important process in plant growth, usually indicating physiological senescence of plants and occurring during different developmental phases or under different biotic or abiotic stresses. The observed phenomenon of Chl degradation is loss of green colour during leaf senescence and fruit ripening. Recently, Chl degradation was demonstrated to occur in green leaves, although this possibility has been suggested for a long time [54]. The requirement for Chl degradation is rationalised by the need to detoxify Chl; thus, senescing cells can sustain viability in order to recycle nitrogen from Chl-binding proteins [55]. In addition, it was suggested that targeting Chl degradation could be a common defense strategy against pathogens and pests in plants [8,56].

**Figure 3:**
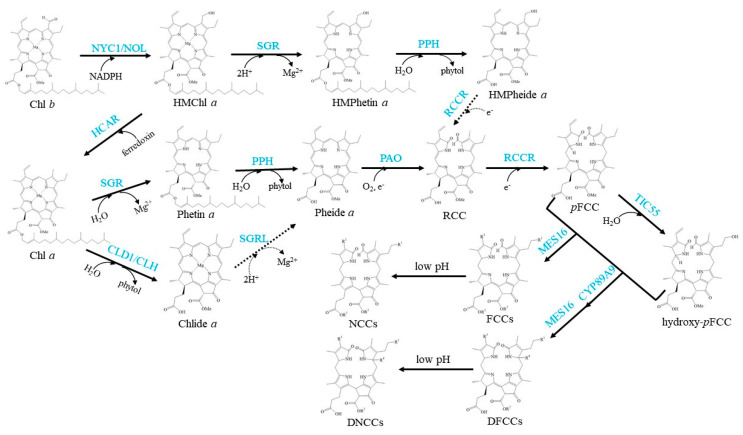
Schemes of the enzymatic reactions in Chl degradation. Arrows show metabolic reactions; Dotted arrows indicate the proposed reactions that need further investigation; Blue characters indicate the enzymes.

The well-established Chl degradation pathway is the so-called Pheide *a* oxygenase (PAO)/phyllobilin pathway (Figure 3).

The conversion of Chl *a* to Phetin *a* is the first step of Chl *a* degradation [57]. In this step, Mg^2+^ is removed from the centre of Chl *a* by magnesium dechelatase, which was sought recently in *Arabidopsis* and encoded by Mendel’s green cotyledon gene *SGR* (*Stay-Green*) [13,58]. *Arabidopsis* SGR family contains two clades: the SGR clade, including SGR1 (also named NYE1 in rice) and SGR2 (also named NYE2 in rice), and the SGR-like clade (SGRL) [59]. Both SGR1 and SGR2 can catalyse the removal of Mg^2+^ from Chl *a* but exhibit low or no activity against Chlide *a*; in contrast, SGRL has higher activity against Chlide *a* [13]. However, none of these three enzymes could remove Mg^2+^ from Chl *b*, implying that Chl *b* cannot directly enter the Chl degradation pathway by Mg^2+^ dechelation [13]. SGR1 plays a crucial role in Chl degradation, and plants lacking SGR1 show a strong stay-green phenotype [60,61]. In contrast, conditional induction of SGR overexpression accelerates green leaf loss [13]. The *in vivo* function of SGR2 is still disputed. It was reported that SGR2 may play negative or positive regulatory roles in Chl degradation during leaf senescence [62,63]. SGRL promotes Chl degradation in different plant species [64,65,66,67]; however, the mechanism needs further investigation.

SGR1 has been suggested to not only function in extracting Mg^2+^ from Chl *a* but may also directly interact with LHCII and be required for recruiting other Chl catabolic enzymes to promote Chl degradation [68]. In contrast, plants lacking SGR1/NYE1 showed a remarkable decrease in the catalytic activity of PAO, another key enzyme in the Chl degradation pathway [61]. In addition, SGR1 can indirectly regulate Chl *b* degradation. Inducing the expression of *SGR* has been shown to accelerate Chl *b* degradation by inducing the accumulation of NYC1, which indicates that a positive feedback pathway exists during Chl *a* degradation function in Chl *b* degradation [69]. This result is reasonable because the Chl *a*/*b* ratio needs to maintain balance in the Chl cycle; when Chl *a* is accelerated to degrade, the degradation of Chl *b* should be enhanced. Recently, it was found that SGR has homologous genes in bacteria lacking Chl [70]. Among these homologues, the Mg-dechelating activities are largely variable. A phylogenetic analysis suggests that a bacterial SGR homologue with high dechelating activity was horizontally transferred to a photosynthetic eukaryote, and SGR acquired substrate specificity after transfer to eukaryotes. Interestingly, a recent investigation showed that SGR is involved in the formation of PSII but not in Chl degradation in *Chlamydomonas reinhardtii* [71].

In the next step, Phetin *a* is converted to Pheide *a* by removing the phytol chain. PPH is the demonstrated Phetin-dephytylating enzyme during leaf senescence [49]. Similar to CLH and CLD1, PPH is also a member of the α/β-hydrolase superfamily, is located in chloroplasts and never accepts Chl as its substrate. The *pph-1* mutant of *Arabidopsis* exhibits a type C stay-green phenotype that retains Chl, but senescence occurs without delay [72]. In addition, the chloroplast membrane and photosynthetic complexes in the *pph-1* mutant remained as the photosynthetic efficiency decreased [49]. In *Solanum lycopersicum* (tomato), *SlPPH*-silenced lines were impaired in Chl degradation and accumulated Phetin *a* during leaf senescence. However, their fruits were able to degrade Chl, similar to wild-type plants [73]. PPH is the major phytol-hydrolytic enzyme during leaf senescence; however, it is not the core hydrolase for Chl degradation during fruit ripening. It has been suggested that CLHs or other unidentified plastid-localising hydrolases may be involved in Phetin dephytylation during Chl degradation in ripening fruits and seed maturation [73].

The porphyrin macrocycle of Pheide *a* should be opened to produce an intermediary product called red-coloured catabolite (RCC) in the next degradation step. This step is the central reaction of Chl degradation, which is catalysed by PAO [74,75]. PAO is a Rieske-type iron-sulfur monooxygenase localised to the inner envelope of mature gerontoplasts. Plants lacking PAO accumulate Pheide *a* and show light-independent and light-dependent cell death [75,76]. It is easy to understand that *pao* knockout mutants showed light-dependent cell death, which because the substrate of PAO, Pheide *a*, is a powerful photosensitizer. Pheide *a* overaccumulation would lead to the accumulation of ROS in chloroplasts under illumination, which causes cell death [76]. On the other hand, the mechanism of light-independent cell death occurring in darkness is still not identified. It was suggested that Pheide *a* specifically inhibits the activity of channel proteins or other cellular components that are essential for membrane integrity, or Pheide *a* functions as a signal molecule that regulates cell death [76].

RCC is a presumably PAO-bound intermediate that is immediately reduced at the C15/C16-double bond by red Chl catabolite reductase (RCCR) to form colourless primary fluorescent Chl catabolites (*p*FCCs), which are detected by their distinctive blue fluorescence [77,78]. Depending on the species, two C16 stereoisomers, *p*FCC and *epi-p*FCC, are formed [79,80]. For example, *p*FCC is formed in *Arabidopsis*, while *epi-p*FCC is formed in *Capsicum annuum* (bell pepper) and tomato [81]. It was demonstrated in vitro that the stereospecificity of *Arabidopsis* RCCR is defined by a single amino acid residue, Phe_219_ [82].

*p*FCCs are modified at several peripheral side positions during normal or species-specific reactions to yield a species-specific set of modified FCCs. Hydroxylation at the C3^2^ ethyl side chain is commonly found in all species analysed [57,83]. This hydroxylation has been demonstrated to occur in the senescent chloroplasts of *Arabidopsis*, and TIC55 is the enzyme that catalyses the hydroxylation of *p*FCC to produce hydroxy-*p*FCC [15]. TIC55 orthologues are phylogenetically distinct from PAO orthologues, and they are widely distributed in higher plants. This indicates that phyllobilin hydroxylation likely appeared with the evolution of land plants.

Both *p*FCC and hydroxy-*p*FCC are exported from chloroplasts by unidentified transporters [57]. They are subsequently further modified in the cytoplasm. Most *p*FCCs and hydroxy-*p*FCCs have been demonstrated to be oxidatively deformylated at the C5-formyl group to produce primary dioxobilin-type fluorescent Chl catabolites (*p*DFCCs) in *Arabidopsis* [84]. This step is catalysed by a cytochrome P450 monooxygenase, CYP89A9, which is located at the endoplasmic reticulum and functions as an FCC deformylase [12]. Catabolite deformylation does not seem to occur in all plant species. For example, this does not occur in *Cercidiphyllum japonicum* [85]. In addition, some FCCs may escape CYP89A9 in *Arabidopsis*. The escaped FCCs can be demethylated to produce O13^4^-demethyl FCCs, which are catalysed by methylesterase family member 16 (MES16) [86]. Again, other species, such as *C. japonicum and Nicotiana rustica*, in which O13^4^ demethylation has not been detected, indicate that they lack an MES16 orthologue [87,88]. DFCCs can also be demethylated to produce O13^4^-demethyl DFCCs; however, further investigation is needed to determine whether this reaction is catalysed by MES16.

Finally, FCCs and DFCCs are imported into the vacuole, and they are nonenzymically isomerized to formyloxobilin-type NCCs and DNCCs respectively in an acidic environment [85]. They are the typical terminal products in higher plants [11,12,89]. Since NCCs and DNCCs were reported for the first time [10,90], many different structures of these two catabolites in different species and tissues have been continuously described [91,92,93,94,95]. Therefore, they are safety to plant cells, and their modification is not conserved and well controlled.

Interestingly, based on the analysis of phyllobilin abundance in cereal and forage crops, it was found that in most analysed grass species, only minor fractions of Chl were recovered as phyllobilins, while phyllobilin quantities match degraded Chl well in *Arabidopsis* [84]. The authors suggested that there is the possibility of Chl degradation beyond the phyllobilin level in grass species. Therefore, other pathway(s) that bypass phyllobilins require further research.

Taken together, we noticed that Chl degradation steps that occur in chloroplasts are relatively conserved, while the steps occur outside of chloroplasts are relatively diversified in plant species. Therefore, removing Chl and its derivatives from chloroplasts to avoid generating too much ROS in chloroplasts should be crucial to plants, and must be well regulated. Because of the same reason, it was hypothesized that stress-related Chl binding polypeptides may function for transient storage of Chl and its derivatives that release from Chl-protein complexes in membranes [96,97]. Once Chl and its derivatives have lost their light-absorbing properties and have been transported out of chloroplasts, they are not able to threat the survival of plants. Therefore, they are degraded through relative diversity pathways, which are species-dependent. This opinion is also supported by the phenotypes of mutant plants which lack the key enzymes respectively [12,34,61,75,86].

## 5. Summary and Open Questions

**Figure 4:**
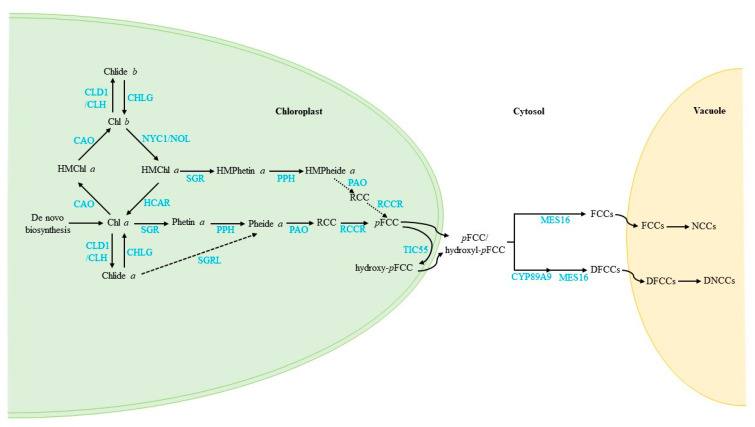
Updated model of the Chl cycle, turnover and degradation pathways in plants. The model shows the subcellular localisation (bold black characters), representative intermediates (black characters) and enzymes (blue characters). Arrows show metabolic pathways; dotted arrows indicate the proposed pathways that need further investigation.

Until now, the Chl cycle, turnover and degradation pathways have been well established in the model plant *Arabidopsis*, as shown in Figure 4.

In short, the interconversion system between Chl *a* and Chl *b* refers to the Chl cycle. During PSII repair, Chls turn over by de- and rephytylation. The major Chl for turnover is Chl *a*. Some Chlide *a* may not be rephytylated if environmental stresses are maintained, and Chlide *a* may join Chl degradation pathways. The PAO/phyllobilin pathway is the major Chl breakdown pathway. Chl is converted to *p*FCC through Chl *b* > HMChl *a* > Chl *a* > Phetin *a* > Pheide *a* > RCC > *p*FCC; next, *p*FCC can be modified at different side positions to produce FCCs and DFCCs, depending on the species. Based on the analysis of an *HCAR*-knockout mutant, the leaf senescence phenotypes showed that Chl can be degraded without HCAR, which implies that HMChl *a* can be degraded directly without conversion to Chl *a*. Therefore, we infer that PAO catalyses HMPheide *a* to RCC. However, further investigation is required to demonstrate this possibility. In addition, other key open questions remain in this research field. First, what is/are the other pathway(s) that bypass phyllobilins in grasses? Second, what is/are the enzyme(s) involved in dephytylation during Chl degradation in ripening fruits and seed maturation? Third, does SGRL catalyse Chlide *a* to Pheide *a* when plants encounter some stress conditions? Fourth, what are the transporters responsible for translocating the intermediates across the organelle membrane?

## Data Availability

Not applicable.

## Abbreviations

CAO	Chlorophyllide *a* oxygenase
CBR	Chlorophyll *b* reductase
Chl	Chlorophyll
CHLG	Chlorophyll synthase
Chlide	Chlorophyllide
CLD1	Chlorophyll dephytylase1
CLH	Chlorophyllase
FtsH	Filamentous temperature-sensitive H protease
HCAR	Seven-hydroxymethyl chlorophyll *a* reductase
HMChl	Seven-hydroxymethyl-chlorophyll
LHC	Light-harvesting complex
MES16	Member 16 of the methylesterase protein family
NCCs	Non-fluorescent chlorophyll catabolites
DNCCs	Dioxobilin-type nonfluorescent Chl catabolites
NOL	NYC1-like
NYC1	Non-yellow coloring1
PAO	Pheophorbide *a* oxygenase
*p*DFCC	Primary dioxobilin-type fluorescent chlorophyll catabolite
*p*FCC	Primary fluorescent chlorophyll catabolites
Pheide	Pheophorbide
Phetin	Pheophytin
PPH	Pheophytinase
PS	photosystem
RCC	Red colored catabolite
RCCR	Red chlorophyll catabolite reductase
ROS	Reactive oxygen species
SGR	Stay-green
SGRL	SGR-like
TIC55	Translocon at the inner chloroplast envelope55
